# Biogenic nanoparticles: overview of challenges in production, characterization and use

**DOI:** 10.3389/fchem.2026.1930144

**Published:** 2026-08-28

**Authors:** Hamidreza Kalantari, Raymond J. Turner

**Affiliations:** 1 Department of Microbiology, Islamic Azad University, Shahr-e Qods, Iran; 2 Microbial Biochemistry Laboratory, Department of Biological Sciences, University of Calgary, Calgary, NW, Canada

**Keywords:** biogenic, biosynthesis, nanomaterial, nanoparticle cap, biocorona, nanoparticles

## Abstract

The biological synthesis of metal-based nanoparticles (NPs) is considered a sustainable and eco-friendly alternative to conventional chemical and physical approaches, offering improved biocompatibility and a reduced environmental footprint. The synthesis and advantages have been highlighted in many studies and reviews; however, the significant challenges continue to limit their broader adoption. This review focuses on these issues within the synthesis, characterization, applications and environment concerns. Limitations such as yield, lengthy and nonuniform synthesis, purification inefficiencies, and high variability between biological sources are discussed. Characterization remains problematic, as biomolecules as capping agents or biocoronas obscure accurate size, morphology, and surface chemistry assessments. Application-specific issues also persist, including instability, inconsistent reproducibility, and uncertainties in long-term safety and toxicity. Environmental risks represent a concern that has been mostly overlooked. These include the potential for bioaccumulation in living organisms, ecological toxicity that can disrupt entire ecosystems, and the current lack of adequate disposal frameworks for nanomaterials. Together, these issues underscore the urgent need for standardized and globally consistent regulatory oversight to ensure the safe development and application of nanotechnology. By critically evaluating challenges, this review aims to guide future research toward the development of robust, reproducible, and safe protocols that will enable the reliable integration of biogenic NPs into medicine, biotechnology, and environmental applications.

## Introduction

A particle of matter with a diameter between one and one hundred nanometers (nm) is commonly referred to as a nanoparticle (Guo et al., 2013). Nanotechnology has influenced and advanced various branches of science. NPs advantages hinge on their high surface area-to-mass ratio, small size, unique chemical and physical properties, and diverse particle morphologies that lead to a manifold of unique properties (Sabet et al., 2016). The use of NPs seems modern but have been used since ancient times without specifically knowing it, such as colorants in the glasses and ceramics (Schaming and Remita, 2015).

Physical characteristics of NPs differ significantly from those of bulk materials, enabling a wide range of novel, unique, and specific NP applications (Hasan, 2015). NPs are typically composed of a uniform molecular core (crystalline or amorphous), capped with compounds for stability and can vary in size and shape, which influences their properties (Hasan, 2015). There are various methods used to produce NPs, which are generally categorized into two main approaches: bottom-up and top-down synthesis (Grappa et al., 2024). Conventional NP production typically involves the use of either chemical or physical techniques, which use either harsh chemicals and/or considerable amounts of energy (Schwarz et al., 2004). Bottom -up uses small chemical precursors and chemical reactions to build a NP; Top-down synthesis is a reductive approach in which larger molecules, metal alloys, or compounds are broken down into smaller and smaller units, eventually to NPs (Khan et al., 2019).

Evolving over the past decade, methods referred to as biological, biogenic, green, or eco-friendly synthesis approaches have gained significant attention in the field because of their inferred nontoxicity and assumed cost-effective nature, as well as their ability to produce NPs with enhanced stability compared with physical or chemical methods (Devi et al., 2024). In this context, studies have focused on synthesizing nanomaterials from biological sources, including herbal plants and plant wastes, algae, seaweeds, viruses, bacteria, and fungi (Vijayaram et al., 2024). The result is a competing field of biologically produced nanomaterials or biogenic NPs versus chemogenic NPs (chemically synthesized).

The properties of chemogenic NPs have been exploited in modern times in biomedical applications for their biocompatibility, anti-inflammatory, antibacterial activities, drug delivery, bioactivity, bioavailability, tumour targeting, and differential biosorption properties (Altammar, 2023). Increased observations have shown that biogenic NPs often exhibit enhanced bioactivities yet the parameters of these NPs in relationship to their bioactivities is only randomly assessed in the literature. Certainly, overall as with chemogenic NPs, biogenic NPs size plays a crucial role, with smaller NPs *typically* demonstrating better efficacy compared with larger ones (Xu et al., 2023).

While biogenic NP synthesis gives alternatives to chemical and physical methods, they present their own challenges, often unique to the biological system used. Observing the field at this state we do not often see studies recognizing all the biological parameters and issues. If they are not appreciated it can result in misleading and improperly interpreted data, reduced yields, and decreased reproducibility.

Biologically synthesized nanoparticles have attracted considerable interest as an alternative to conventionally synthesized nanoparticles because they aim to combine principles of green chemistry with the unique capabilities of biological systems (Kirubakaran et al., 2026). In contrast to many chemical and physical synthesis methods, biological synthesis is generally performed under mild reaction conditions and often eliminates or reduces the need for hazardous reagents. In addition to potentially lowering the environmental footprint of nanoparticle production, biological systems can simultaneously reduce metal ions and stabilize the resulting nanoparticles through naturally occurring biomolecules (Devi et al., 2024). Unlike chemically synthesized nanoparticles, which commonly require synthetic surface modifiers to achieve stability or functionality, biogenic nanoparticles inherently acquire a biomolecular capping layer during synthesis (Sidhu et al., 2022). This naturally formed interface not only contributes to nanoparticle stability but also influences their interactions with biological systems, offering opportunities for biomedical and environmental applications. Nevertheless, these same biological components introduce additional complexity, making reproducibility, purification, characterization, and standardization more challenging than for conventionally synthesized nanoparticles. Understanding this balance between the unique advantages and the associated limitations is therefore essential for the continued development and practical application of biogenic nanomaterials.

In this review, we focus on the challenges associated with biogenic nanoparticles while also assessing the advantages that make biological synthesis an attractive alternative to conventional chemical methods. Particular emphasis is placed on the production, purification, characterization, and applications of biologically synthesized metal-based nanoparticles, with the aim of identifying current limitations and discussing approaches to improve the reliability, reproducibility, and standardization of biological synthesis. A major focus of this review is the biomolecular capping layer, one of the defining characteristics that distinguishes biogenic nanoparticles from chemically synthesized counterparts. Understanding the composition and role of this biomolecular cap, together with the subsequent formation of the biocorona in biological environments, is essential for the accurate characterization, rational design, and successful application of biogenic nanoparticles. In addition, considerations are indicated regarding the life cycle of biogenic nanomaterials, including their production, use, and environmental implications, and their safe and reliable implementation(s).

### Why biogenic nanoparticles?

Although chemical and physical methods have enabled the controlled production of nanoparticles for decades, growing environmental concerns together with the increasing demand for sustainable manufacturing have stimulated interest in biological synthesis approaches (Jain et al., 2024). Biogenic nanoparticles are typically produced under relatively mild reaction conditions using biological systems or their biomolecules (Abdallah et al., 2026). Unlike chemically synthesized nanoparticles, which often require inorganic reducing agents and surface functionalization, biogenic synethesis supplies its own reducing chemistry and the resulting nanoparticles naturally acquire a biomolecular capping layer during synthesis (Durán et al., 2023). This biological NP surface interface not only contributes to nanoparticle stability but also influences cellular interactions, biological activity, and physicochemical properties (Wang et al., 2019).

These distinctive characteristics have generated considerable interest in biomedical, environmental, and industrial applications, including antimicrobial therapy, drug delivery, cancer treatment, biosensing, and catalysis. However, many of the same features that make biogenic nanoparticles attractive also introduce challenges. The heterogeneous composition of the biomolecular capping layer, variability among biological sources, difficulties in purification, and limited mechanistic understanding can affect reproducibility, characterization, scalability, and regulatory acceptance (Lakshmikanthan et al., 2026). Consequently, although biogenic synthesis offers several potential advantages, its successful translation into practical applications depends on addressing these challenges through improved mechanistic understanding, standardized methodologies, and rigorous characterization.

Some key similarities and distinguishing characteristics of chemically synthesized and biogenic nanoparticles are summarized in Table 1, providing the framework for the discussions throughout this review.

**TABLE 1:** Comparison of chemically synthesized and biogenic metal nanoparticles highlighting their respective advantages and current challenges**.**

Parameter	Chemically synthesized nanoparticles	Biogenic nanoparticles	Current challenge/perspective
Reducing and stabilizing agents	Chemical reducing agents and synthetic stabilizers provide precise reaction control	Natural biochemistry simultaneously reduce and stabilize nanoparticles, eliminating or reducing the need for additional synthetic stabilizers	Identifying the biomolecules responsible for reduction and stabilization mostly overlooked and remains a research priority
Surface capping layer	Surface ligands are intentionally selected and chemically defined	Nanoparticles acquire a naturally formed biomolecular capping layer that is more often heterogeneous in composition	This natural capping layer may improve biological interactions and functionality, but its composition and molecular organization remain poorly understood
Reaction conditions	May require hazardous reducing agents, solvents, elevated temperatures, pressures and energy depending on the synthesis route	Often performed under mild aqueous conditions using renewable biological materials. Have their own unique energy and hazard considerations	Overall sustainability should consider the complete production process, including extraction, purification, energy consumption and waste generation
Particle size and morphology	Generally allows tight control over size and morphology	A wide range of particle sizes and morphologies can be achieved through biological systems, although variability and control are often a challenge	Better understanding of biological reaction mechanisms could improve control
Surface functionality	Frequently requires post-synthesis functionalization for biomedical applications	Naturally occurring surface biomolecules may provide intrinsic bioactivity and facilitate further biological interactions or give it biohazard properties	The specific contribution of the biomolecular cap to biological performance remains insufficiently understood
Biocompatibility	Often improved through additional surface modification	Frequently reported to exhibit improved biocompatibility because of naturally occurring surface biomolecules	Long-term *in vivo* studies are still needed to determine whether these advantages are consistent across different biological systems
Purification	Primarily removes unreacted chemicals and synthetic reagents providing pure NPs	Requires removal of residual biological materials while preserving the functional biomolecular capping layer. Typically less pure and/or homogenous	Standardized purification protocols capable of distinguishing functional capping molecules from contaminants are still lacking
Characterization	Surface chemistry and physical properties are generally easier to interpret.	Characterization is complicated by heterogeneous biomolecular coatings and residual biological materials	Complementary analytical methods are required to fully characterize both the nanoparticle core and its biological interface
Reproducibility	Usually high under standardized reaction conditions	Influenced by natural and systematic biological source variability	Developing standardized biological synthesis protocols
Biomedical applications	Several formulations have progressed to commercial or clinical use	Rapidly growing evidence supports applications in antimicrobial therapy, drug delivery, cancer treatment and vaccine development	Better understanding of the biomolecular cap may improve targeting efficiency, stability and clinical translation
Environmental considerations	Environmental impact depends largely on chemical reagents and waste management. Disposal tends to still be overlooked	Reduced use of hazardous chemicals makes biological synthesis an attractive alternative. Yet disposal is overlooked	Environmental safety should be evaluated throughout the nanoparticle life cycle rather than considering synthesis alone

## Biological synthesis of nanoparticles

**FIGURE 1:**
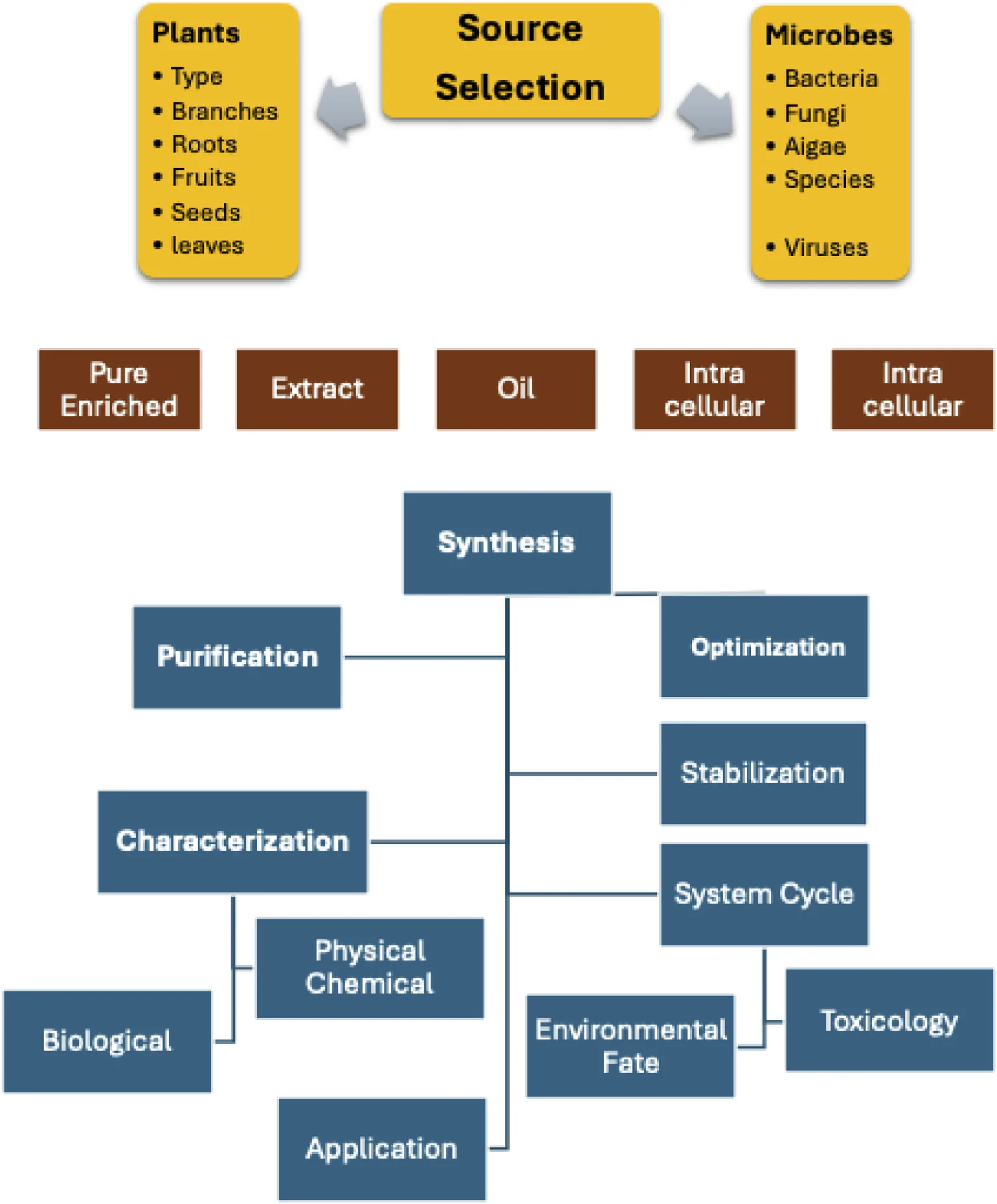
Schema of steps involved in Biogenic synthesis process towards stable characterized nanoparticles for applications.

In the field biogenic synthesis of NPs, a wide range of biological systems have been explored as catalysts, including those from plants, bacteria, algae, viruses, and fungi (Figure 1) (Anwar, 2018; Kumar and Yadav, 2009). These biological systems have been successfully used to synthesize various metal- and metal oxide-based NPs, such as silver (Ag), gold (Au), selenium (Se), tellurium (Te), and various mixed atom nanoparticles such as zinc oxide (ZnO), copper oxide (CuO), or iron oxide (Fe_3_O_4_). Depending on the biological source and synthesis conditions, the resulting NPs typically range from 5 to 100s of nm in diameter/length, (Khan et al., 2026; Kirubakaran et al., 2026). In addition to the use of extracted biomolecules as catalysts for the required redox reactions, extracts, whole cells or parts of living organisms may also function as biofactories, where intracellular or extracellular biomolecules mediate redox chemistry towards NP formation (Choi and Lee, 2020). The choice of biological system significantly influences the synthesis rate, particle size, morphology, yield, and the composition of the biomolecular capping layer, all of which ultimately affect the physicochemical and biological properties of the synthesized NPs (Sidhu et al., 2022).Although these factors are often discussed independently, they largely originate from a common source: variability in the biochemical reaction environment. Differences in plant or microbial species, genotype, growth conditions, developmental stage, extraction procedures, pH, temperature, ionic composition, and the abundance of reducing metabolites and/or enzymes can alter the chemical environment in which metal ions are reduced. These variations influence metal-ion speciation, redox kinetics, nanoparticle nucleation and growth (diffusion properties of atoms in the biological ‘soup’), aggregation, and ultimately the composition and structure of the biomolecular capping layer. Consequently, many of the challenges observed, including variability in particle size, morphology, stability, reproducibility, and biological activity, should be viewed as interconnected outcomes of changes in the biochemical reaction environment rather than as isolated phenomena. Despite considerable progress in biogenic nanoparticle synthesis, the molecular mechanisms linking these biological variables to nanoparticle formation remain only partially understood, highlighting an important area for future investigation.

### The surface of nanoparticles

**FIGURE 2:**
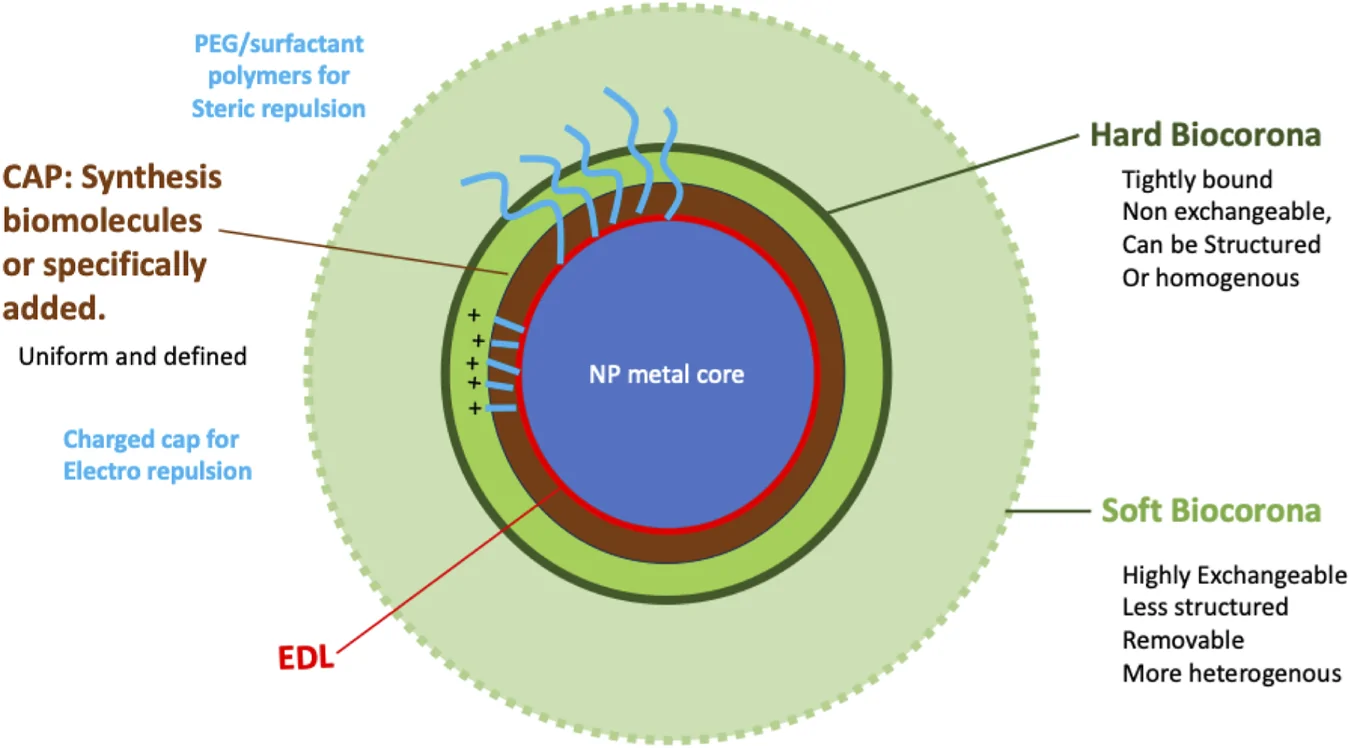
Representation of a NP with various layers over the metal core. The immediate surface generates an electrical double layer. In chemoNPs the next layer is specifically added molecules for repulsion for stabilisation (cyan). In the case of biogenicNP the immediate cap would be biomolecules that may or may not have been part of the NP synthesis process but would be part of the cell or cell extract used (brown layer. This layer would then interact with other biomolecules generating the Hard biocoronal layer, (green) and the effective size of the NP. Yet this layer will also attract and loosely interact with other biomolecules generated the Soft biocorona. (PEG: Polyethylene glycol).

A key distinguishing feature between chemo and biogenic NPs is the surface of the NP. The chemistry of the surface layer, the capping or coating of the NP, is within the control of the chemist, but becomes a function of the biochemistry of the organism for biogenic NPs. Therefore, here it is important to make a distinction between the biomolecular cap, which originates from plant or microbial compounds during synthesis, and the idea of a biocorona (Shannahan, 2017), which forms when chemogenic NPs interact with biological environments. The surface of the metal core can be described through multiple layers from the surface (Figure 2).

**FIGURE 3:**
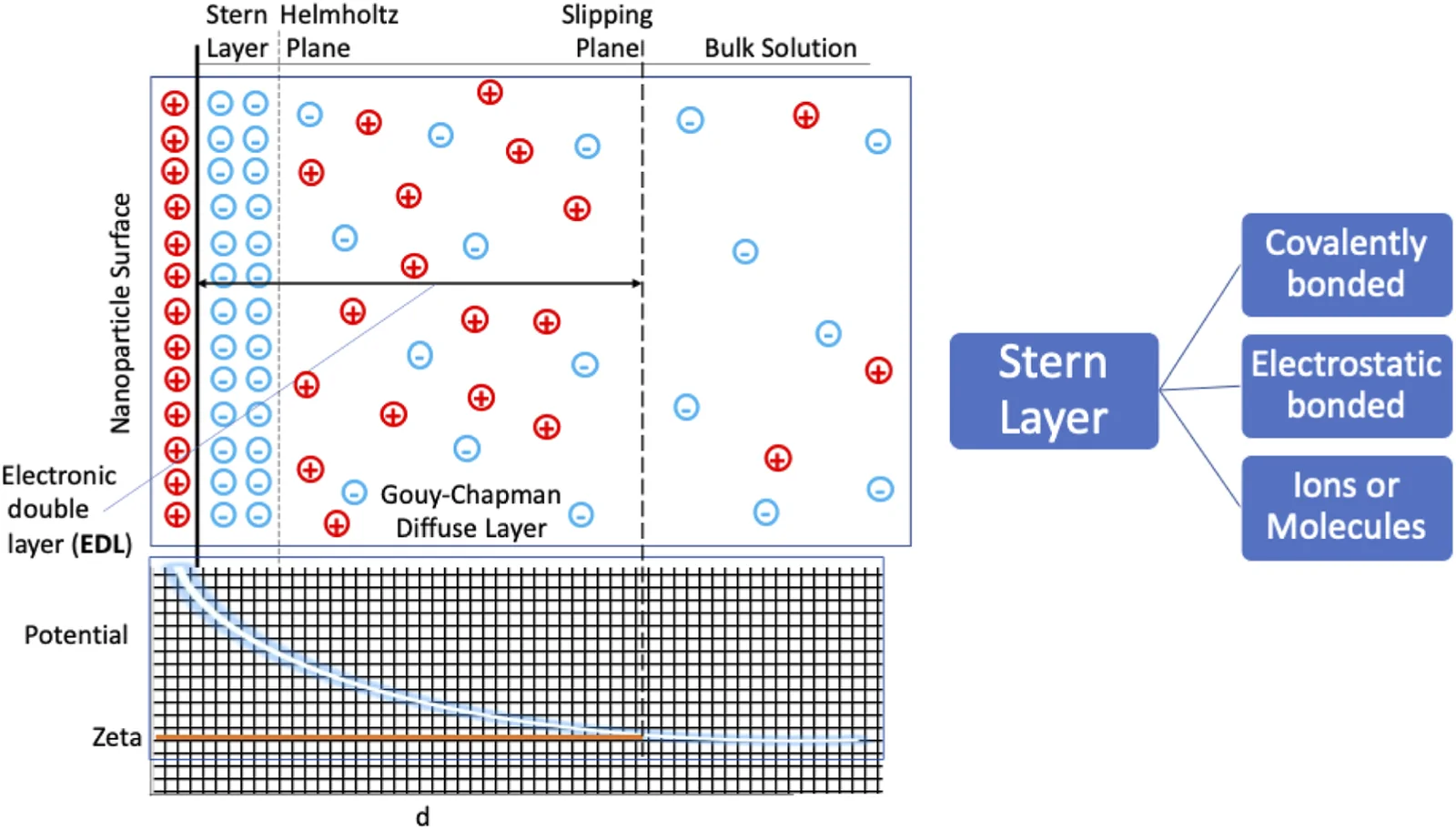
Physical chemistry description of the surface of a metal nanoparticle. The figure reflects the various layers of charge and its relationship to the zeta potential measurement. Biomolecules and/or ions can be part of the Stern layer and the Diffuse layer which may be covalently attached to the NP metal surface or interacting via electrostatics. Regardless, the net charge at the slipping plane is considered a measure of the stability of the NP in regard to either further metal atom addition or two particles coming close enough together to fuse into larger particles.

In the sense of chemoNPs, specific chemicals are added to the surface for thermodynamic stabilization via steric or electro repulsion. These chemicals are covalently attached and/or tightly bound. This chemical layer is well defined and quite uniform. In the case of biogenicNPs the cap consists of biomolecules from the organism that was used. These biomolecules are typically very tightly associated similar to chemoNP caps and are exposed beyond the surface electric double layer (EDL) (see also Figure 3). This biogenic cap can be heterogenous in type of molecule and less structured. Beyond this tight cap one also acquires a biocorona. The biocorona are biomolecules that interact with surface capping molecules that and become associated as part of the superstructure of the NP leading to several layers in its surface (Akhter et al., 2021; Wheeler et al., 2021) (Figure 2).

The biocorona typically has a tightly associated layer, the Hard (*ex vivo* corona). In the case of biogenic this would be the biomolecules that adsorbed to the surface during the biogenic synthesis. In the case of biogenic synthesis using defined set of biomolecules (proteins, carbohydrates, cofactors), this corona often contains the catalytic molecules as a well-defined biomolecule layer. In the case of using cells or extracts the NP is formed in the ‘soup’ of defined biology and the hard corona would be more diverse in composition. This layer is tightly interacting with the surface of the NP core or at the EDL and typically these molecules do not exchange. The biomolecules of this corona are solely from the biology of the biogenic synthesis. This corona may also form with a defined structure or lattice. In some cases, this hard corona may be part of the Diffuse layer and influence the zeta potential. Additionally, there is often a Soft Corona (*in vivo* corona). This corona may form in complex biological synthesis systems but is also obtained when the biogenic nanoparticle is functionally used in biological systems (drug delivery, as antimicrobial). One can also have an environmental or eco-corona from other environments (aquatic, marine, soil, WWTP) where inorganics and non-biological molecules become part of the corona. This soft corona contains molecules that are less tightly bound, less structured, more diverse, easily exchanged and easily desorbed and removable through purification.

Both the initial biomolecular capping and the biocorona are of course influenced by the type of organism, its growth conditions, and subsequent use, which determine the molecular composition surrounding the NPs (Soliman et al., 2024) and thus the stability and functionality of the biogenic NPs (Jain et al., 2024).

### Plants

Biomolecules from plants, such as enzymes, redox proteins, flavonoids, sterols, terpenes, terpenoids, saponins, cofactors and various other alkaloids, which can act as redox agents and found as NP capping molecules. These biomolecules participate in the chemical transformation of metal ion precursors through reduction, oxidation, hydrolysis, precipitation, or biomineralization processes, depending on the chemistry of the metal system. These reactions ultimately lead to the nucleation and growth of nanoparticles while simultaneously contributing to the formation of the biomolecular capping layer. (Raja et al., 2017; Vijayaraghavan et al., 2012). In the synthesis of NPs, various plant parts, such as leaves, flowers, stems, roots, rhizomes, bark, seeds, and peels, have been utilized as sources to prepare extracts. For example, a developed facile protocol for synthesizing gold NPs from ginger root was shown to exhibit properties compared with those of chemically produced ones (Kalantari and Turner, 2024). Additionally, the Plant biogenic synthesis approach, the use of plant extracts offers several advantages over microbial methods. Notably, it eliminates the need for isolating or cultivating microbial cell cultures, as well as the complex separation of bacteria cells from the synthesized NPs, making the process more straightforward and cost-effective (Ahmed et al., 2016; Sharma et al., 2019).

In addition to terrestrial plants, algae (including both macroalgae and microalgae) have emerged as an attractive biological source for NP synthesis (Chaudhary et al., 2020). Algal extracts are rich in polysaccharides, proteins, pigments, polyphenols, and other metabolites that act as reducing and stabilizing agents, enabling the synthesis of a wide range of metal and metal oxide nanoparticles under mild reaction conditions (Cadar et al., 2025). Compared with terrestrial plants, algae are often more useful because of their rapid biomass production, ease of cultivation, and the absence of competition with agricultural land, making them promising candidates for sustainable large-scale nanoparticle production (Laura, 2025). Algae-mediated synthesis has gained considerable attention because algal biomass can be cultivated in freshwater or marine environments with relatively low resource requirements compared to microbial cultivation (Sarwer et al., 2022). Recent studies have demonstrated the successful synthesis of gold, silver, zinc oxide, iron oxide, and other nanoparticles using green, brown, or red algae, further expanding the diversity of biological systems available for nanoparticle production (Hameed et al., 2023; Laura, 2025).

Regardless, the use of plants for NP synthesis faces several issues, including geographical limitations regarding a given plant species survival ecosystem, issues with reproducibility, feasibility for mass production, and the unexplored influence of soil or water variation. Some examples of these challenges are summarized in Table 2. Examples of different plants and plant materials used for the biogenic production of some common NPs are presented in this Table gives sections for gold, silver, copper and zinc oxide biogenic NPs. Each section in Table 3 highlights some of the issues associated with using a particular plant species for the respective metal NPs biosynthesis.

**TABLE 2:** Examples of issues in using plants to produce nanomaterials.^#^

Plant Name	Geographical Limitation	Reproducibility	Mass Production Feasibility	Influence of Soil Variation	References
*Butea monosperma*	Native to South Asia	Moderate	Moderate	Not studied	Lagashetty (2022), Patil et al. (2023)
*Rubus sp*. (Blackberry)	Widely available but varies by species	Low	Low	Not studied	Tasić et al. (2024)
*Crassocephalum rubens*	Native to Central Africa, introduced to China, Myanmar, and Thailand	Low	Low	Not studied	Adewale et al. (2023)
*Ocimum basilicum* (Purple Basil)	Grown in various regions, but chemical composition varies	Moderate	Moderate	Significant impact on chemical composition and NP properties	Solmaz et al. (2024), Ünal and Aydoğdu (2024)

^#^ As identified within gold and silver biogenic NP production studies. Moreover, The qualitative assessments presented in this table (e.g., low, moderate, high, unclear, and not studied) represent the authors’ evaluation of the available evidence rather than terminology used by the original publications. Classifications were assigned based on the information reported in each study, including experimental design, discussion of reproducibility, scalability, purification, characterization, and environmental or practical limitations, where applicable. When a particular aspect was not directly investigated or discussed in the original study, it was classified as “not studied” or “unclear” rather than inferred.

**TABLE 3:** Examples of plant-mediated synthesis of metal NPs.

Plant Source	Challenges in synthesis	Challenges in characterization	Challenges in application	Environmental concerns	References
Gold nanoparticles (AuNPs)					
Neem (*Azadirachta indica*)	Variation in phytochemical composition affects reproducibility	Unclear SEM images and low UV absorption rate due to unpurified biological compounds	Poor long-term stability	Unclarified	Anuradha et al. (2010), Prashanth and Krishnaiah (2014)
Green Tea (*Camellia sinensis*)	Limited stability in distilled water may cause NP agglomeration or property changes over time	Plant metabolites interfere with SEM and FTIR spectra	Rapid aggregation in biological fluids affects medical applications	Potential toxicity to aquatic life in high concentrations	Pirko et al. (2021)
Aloe Vera (*Aloe barbadensis miller*)	High viscosity of plant extract complicates NP formation	Overlapping absorption peaks in UV‒Vis due to plant biomolecules	Limited stability	Potential toxicity remains unstudied	Kumari et al. (2022a)
Turmeric (*Curcuma longa*)	Unclear plant collection time, source, and material state	Aggregation observed in TEM images	Effective antimicrobial activity, but the influence of biological compositions and pH remains uncertain	Uncertain potential activity of aggregated NPs	Dharman et al. (2021)
Mango (*Mangifera indica*)	Alcohol-based solvents were used without reasons instead of water	Difficult to separate gold NPs from plant residues	potential for diabetes treatment, but the roles of the biological compositions are unclear	Unknown long-term effects of plant-derived AuNPs on human body	Badeggi et al. (2024)
Garlic (*Allium sativum*)	High sulphur content may lead to excess capping and aggregation issues	Difficult to understand the capping layer	Shows antifungal activity, but the effect of sulfur remains unclear	Sulfur interaction with gold may alter environmental fate	Jikah et al. (2024), Umamaheswari and Abirami (2023)
Basil (*Ocimum sanctum*)	pH sensitivity affects NP formation and reproducibility	Difficulty in distinguishing plant biomolecules from gold peaks in UV‒Vis	Not suitable for high-temperature applications due to organic coating	Nontoxic, but its kidney absorption and excretion remain unclear	Nayak et al. (2022)
Silver nanoparticles (AgNPs)					
Neem (*Azadirachta indica*)	This method results in heterogeneous NPs	Presence of plant metabolites interferes with UV‒Vis and FTIR spectra	Shows good anticancer potential, but side effects remain unclear	Bioaccumulation in soil and water may affect microbial diversity	Kumari et al. (2022b)
*Scabiosa atropurpurea*	Geographically limited	The FTIR shows bio cap, but not accurate due to insufficient purification	Good inhibitory activity against bacteria but not a good bactericidal	Noncytotoxic at low concentrations (dose-dependent)	Essghaier et al. (2022)
*Calotropis procera*	Geographical limitation of this plant hinders reproducibility	Large NP size (90–100 nm), near the nanoscale boundary	Effective antimicrobial activity against *Vibrio cholerae*, but the role of the capping layer remains unclear	Toxicity is not clear	Salem et al. (2015)
*Mimusops elengi*	Rare and geographically limited availability	Organic molecules interfere with precise NP sizing in DLS	Surface instability limits use in medical applications	Ecosystem impact remains unstudied	Prakash et al. (2013)
Mango (*Mangifera indica*)	Difficulties in maintaining consistent NP morphology	Presence of flavonoids affects FTIR interpretation	Poor stability in biomedical applications	Unknown long-term effects on plant‒soil interactions	Hai et al. (2022)
Garlic (*Allium sativum*)	Sulfur-containing compounds may lead to excessive capping	Strong organic interactions interfere with SEM imaging	Different antibacterial effectiveness	Silver NP accumulation in water may harm marine life	Gabriel et al. (2022), Shafea and Mahmoud (2021)
Basil (*Ocimum sanctum*)	Sensitivity to pH variations impacts nanoparticle synthesis	Difficult to separate plant-derived organic residues from AgNPs in characterization	Limited efficacy in medical applications due to inconsistent particle size	May alter natural microbial communities in the environment	Gautam et al. (2023), Qaeed et al. (2023)

Plant Source	Synthesis Conditions	Applications	Challenges	References
Copper nanoparticles (CuNPs)				
Garlic (*Allium sativum*)	Aqueous extract, CuSO_4_ as precursor	Antibacterial, antifungal, wound healing	High oxidation rate reduces stability, aggregation in solution	Kumar et al. (2024)
Black Pepper (*Piper nigrum*)	Aqueous extract, Cu(NO_3_)_2_	Antimicrobial, antioxidant	Polydispersity in size, difficulty in purification	Shafiq et al. (2024)
Clove (*Syzygium aromaticum*)	Aqueous extract, CuCl_2_	Potential pharmacological and industrial applications	Leads to heterogeneous NPs	Elbagory et al. (2022)
Zinc oxide nanoparticles (ZnO NPs)				
Moringa (*Moringa oleifera*)	Aqueous extract, ZnSO_4_	Antibacterial	Requires high-temperature calcination	Irfan et al. (2021), Ogbeide et al. (2025)
Green Tea (*Camellia sinensis*)	Aqueous extract, Zn(NO_3_)_2_, mild heating	Antioxidant, antibactericidal and anticancer	Low yield, aggregation in aqueous medium	Al-Ghamdi et al. (2022)
Banana Peel (*Musa paradisiaca*)	Zn(NO_3_)_2_ precursor, hydrothermal method	Antimicrobial against *Staphylococcus epidermidis*	Requires additional processing for stability	Al-Khaial et al. (2024), Apriandini et al. (2022)

A key factor is the accessibility and availability of a given plant. Many of the plants used in NP synthesis are limited to specific geographical regions, making it difficult to reproduce experiments in different climates. For example, Adewale (Adewale et al., 2020) used *Crassocephalum rubens* to synthesize gold NPs. According to data from the Plants of the World Online (POWO; powo.science.kew.org), this plant is native to Central Africa and has minor introductions in China, Myanmar, and Thailand limiting accessibility to these Asian areas. Such geographical restrictions can pose challenges for global applicability.

Seasonal variations can significantly influence the biochemical composition of plants, including their protein content and other phytochemicals. These fluctuations can be important in NP synthesis, as the timing of plant material collection can impact the efficiency and characteristics of the NPs produced and what biomolecules are incorporated as part of the final NP. Variability in phytochemical composition may affect key synthesis parameters leading to variations in particle size, shape, and stability, potentially leading to inconsistencies in experimental outcomes (Bıçakçı and Türk, 2024).

Additionally, a factor similar to seasonal issues is the variation in the biomolecular composition of the same plant species when it is grown in different soil types. Soil characteristics, including nutrient content, pH, texture, and microbial communities, can significantly influence a plant’s chemical profile, such as its essential oil content, protein levels, and metabolite composition. For example, a study has shown that *Ocimum basilicum* (purple basil) exhibits remarkable compositional differences depending on the soil conditions (Tursun, 2022). In a 2017 study by Malapermal et al., this plant was used for the synthesis of silver NPs (Malapermal et al., 2017). However, if the same plant is grown in a different soil environment, variations in its phytochemical composition led to differences in NP properties. Collectively, the examples indicate that the challenges associated with plant-mediated nanoparticle synthesis are largely independent of the plant species employed. Instead, they arise from common factors inherent to biological systems, including variability in phytochemical composition, geographical and seasonal influences, differences in extraction procedures, and limited process standardization. Consequently, although numerous plant species have been successfully used for nanoparticle synthesis, improving reproducibility depends less on identifying new plant sources than on developing standardized extraction, synthesis, and purification protocols (Syed et al., 2026a; Syed et al., 2026b).

### Microorganisms

Microbial biogenic NP production has the advantages over plants as there is no geographical or geological limitations. Yet similar to the use of plants: which microbe would be the most effective for the synthesis of a given metal NP? This remains a subject of debate and is often influenced by researcher bias and the intended application.

Various fungi and yeasts have been explored for their ability to synthesize NPs (Pradhan and Turner, 2023). For example, research conducted with *Yarrowia lipolytica* revealed that it is a candidate for synthesizing selenium NPs (Lashani et al., 2024). Other yeast species, such as *Saccharomyces*, *Magnusiomyces*, and *Rhodotorula,* have also been utilized for NP production, highlighting the potential of yeast-based approaches in eco-friendly nanotechnology (Ashengroph and Tozandehjani, 2022; Ruocco et al., 2014; Salem, 2022). Additionally a variety of bacterial strains have been employed to produce various metal NPs (Arora et al., 2024). An advantage of using microbes is that the biogenic production could be via biotechnology large-scale fermenters to overcome the environmental and climate considerations for plants. This microbial approach offers a sustainable and eco-friendly alternative for NP production, further expanding the potential of biological synthesis methods (Azizi and Daneshjou, 2024; Hajiali et al., 2025; Pallath et al., 2024; Wei and Zheng, 2024). Table 4 presents a brief list of examples of microorganisms used for NP synthesis, along with the associated challenges identified across studies.

**TABLE 4:** Examples of Microbe-mediated nanoparticle synthesis and related challenges.

Microorganisms	NPs	Production	Applications	Challenges	References
*Lactobacillus casei*	Ag	Intracellular	Plant-growth stimulator, antimicrobial antifungal	Purification is energy-intensive and often incomplete	Singh et al. (2015)
*Actinobacter* spp	Au	Intracellular	Antimicrobial antifungal, nano fertilizer	Heterogeneous NPs (5–500 nm), slow growth rate, and complex purification processes	Bharde et al. (2007)
*Staphylococcus aureus*	Ag	Extracellular	Plant-growth stimulator, antifungal antimicrobial	Leads to very large NPs with potential biosafety risks	Nanda and Saravanan (2009)
*Bacillus licheniformis Dahb1*	Ag	Extracellular	Antibiofilm	Exhibits antibiofilm activity at high doses, but achieving such concentrations is challenging	Shanthi et al. (2016)
*Escherichia coli k12*	Au	Extracellular	Biocatalyst in complete reduction of nitroaromatic pollutant in water	High cost and low yield make this method unsuitable for treating nitroaromatic pollutants in water	Srivastava et al. (2013)
*Lactobacillus kimchicus DCY51T isolated from Korean kimchi*	Au	Intracellular	Antioxidant, drug delivery systems, cancer diagnostic	Incomplete purification may retain intracellular toxins like LPS, potentially capping the NPs	Markus et al. (2016)
*Lactobacillus acidophilus DSMZ 20079T, Bacillus subtilis ATCC 6633*	CdS	Extracellular	Nanobiosensors	Random aggregation of CdS NPs within the PHB matrix may affect uniform dispersion and material properties	Riaz et al. (2020)
*Lactobacillus acidophilus* *Lactobacillus casei* *Bifidobacterium* sp	Se	Extracellular	Plant disease enhancer Nanofertilizer	Large NP size (100–500 nm), limiting nanoscale benefits Potential toxicity concerns due to selenium overdose	Eszenyi et al. (2011)
*Lactobacillus casei*	Cu	Extracellular	Plant micronutrient Anticancer Antimicrobial	potential cytotoxicity, unclear stability, variability in biosynthesis	Kouhkan et al. (2020)
*Streptomyces griseus*	Cu	Extracellular	Nanobiofungicides	unclear environmental impact, toxicity at high doses	Ponmurugan et al. (2016)

A feature of using bacteria, is that their bioenergetic systems are at the cell surface. Taping these energy systems leads to bacteria producing NPs associated with these energized membranes either extracellularly or intracellularly, depending on the metal’s physicochemical properties but also the bacterial species. The environments between intracellular (within the cytoplasm) or extracellular leads to different chemical environments of pH, redox potential, ionic strength, ions and biomolecule types and concentrations. These different environments defines that the metal ion will have different speciation states: their geometries, redox, charge state, counter ions and types of ligands and degree of hydration from (H_2_O, H_3_O^+^, OH). For NP synthesis to occur is the combination of the specific chemical reaction areas in the cell and the appropriate metal speciation. The outcome gives differences in the final NP shape, size, cap composition as well as the yield and how easy it will be to purify and scale up.

A consideration in the choice of microbial species is the biomolecules that NPs may retain from their biogenic production hosts. Therefore, NPs synthesized from pathogenic bacteria may carry immune-responsive biomolecules or even toxins. Biogenic NPs synthesized by bacteria, fungi or viruses could decorate their NP surfaces with potentially harmful proteins and biomolecules. These biomolecules that remain associated with these biogenic NPs, typically with their cap or shell, influence their shape, stability, and functional attributes, which influence their biomedical and/or industrial applications.

One of the major challenges of using microorganisms for NP synthesis is their small-scale production and potentially lengthy incubation process, which depend on factors such as the type of bacteria or fungi, culture conditions, and incubation temperature. Typically, microbial synthesis takes hours. This time encompasses the culturing of the cells to high density to provide a high concentration of cells. Production of NPs may occur during the culture growth or after culture meets its appropriate maturity at which metal salts are added at a given point in the growth cycle.

Another consideration is the potential safety risks associated with certain bacterial strains. For example, pathogenic bacteria such as *Salmonella typhimurium*, *Klebsiella pneumoniae*, and *Bacillus anthracis* have been utilized in NP synthesis (Banerjee et al., 2021; Ghorbani et al., 2015; Malarkodi et al., 2013). However, the potential of immune-responsive bacterial biomolecules in the final NP product remains unclear and is often untested. Additionally, some bacteria pose biological hazards and be infectious leading to disease, raising concerns about whether such synthesis methods can truly be considered safe and environmentally friendly (Table 5).

**TABLE 5:** Challenges of using microorganisms for synthesis of nanoparticles^#^.

Challenges	Descriptions
Low Production Scale	Microbial synthesis is often limited to small-scale production, making it difficult to achieve mass production for industrial applications
Long Synthesis Time	The process typically takes longer than chemical synthesis, requiring incubation times that vary based on microbial type, culture conditions, and temperature
Variability in Culture Conditions	Factors such as nutrient availability, pH, temperature, and incubation time can significantly influence NP yield, size, and stability
Pathogenicity Risk	Some bacteria used in synthesis, such as *E. coli, P. aeruginosa, S. typhimurium, K. pneumoniae,* and *B. anthracis*, pose health hazards and require biosafety precautions
Immune Response Concerns	NPs synthesized by bacteria may carry immune-responsive biomolecules, raising questions about their biocompatibility and potential immunogenic effects
Biosafety and Regulatory Issues	The use of genetically modified or pathogenic microorganisms requires strict biosafety regulations, making large-scale implementation challenging
Reproducibility Issues	Differences in microbial strains, culture media, and environmental conditions can lead to variations in NP properties, affecting consistency and standardization
Purification Challenges	Removing microbial residues from the final NP product can be complex and may require additional purification steps to ensure safety and stability

^#^ As identified from the broader literature on microbial biogenic NP production.

Overall, microbial synthesis offers potentially better control through defined culture conditions than plant-based approaches; however, it introduces its own challenges, including strain-dependent variability, differences in metabolic activity between cultures, and complex downstream purification. These limitations suggest that successful scale-up will depend on control of biological processes rather than simply selecting different microbial species for different NP types.

## Challenges in studying biogenic NPs

### Reaction mechanism

The exact chemical synthesis mechanisms of biogenic NPs tend to be difficult to fully dissect. Although the precise molecular pathways vary among biological systems, biogenic nanoparticle synthesis is generally considered to involve the reduction of metal ions, followed by the nucleation and growth of metal atoms into nanoparticles, and their stabilization by surrounding biomolecules. The relative contribution of each of these processes depends on the biological source, the metal precursor, and the reaction conditions. This challenge is highlighted in the discussion of Se and Te oxyanion physiology (Kessi et al., 2022), where there are many pathways of reduction towards Se or Te NPs for these two related elements, that with decades of studies, their remains many unknowns. Certainly, each organism used will have different and diverse biomolecules that may be involved in the biogenic synthesis. Certainly hard-soft acid base theory and redox chemistry is involved (Lemire et al., 2013). In general, electron-donating biomolecules or enzyme-mediated redox reactions are believed to initiate metal ion reduction (Shivani et al., 2026). The resulting metal atoms subsequently nucleate and grow into nanoparticles in the localized environment around the reductant, while biomolecules present in the reaction medium may adsorb onto the nanoparticle surface, contributing to particle stabilization, size and shape and influencing the composition of the biomolecular capping layer (Ahmad et al., 2022). But the specific biomolecules involved in the reactions can be difficult to discern. Suggested candidates include reductase enzymes using reductive energy such as NAD(P)H, heme, flavins, carotenoids, or cytochromes. Where the enzymes reduction potential and the metal species give a favourable free energy allowing the reaction to occur via the metal high-jacking or diverting a biochemical reaction. Another candidate includes free thiols (R-SH) from cysteine amino acids in peptides (e.g., glutathione) or enzymes. Other candidates include other reactive functional group sources, particularly from plants one must consider reducing sugars, (either free or in cellular polymers), alkaloids and polyphenols. The challenge is reflected in using banana peels in the synthesis of AgNPs which has been done for over 20 years, yet reports are still adding to what biomolecules may be carrying out the reaction(s) (Goh et al., 2023).

Taken together, current evidence suggests that biogenic nanoparticle formation cannot be attributed to a single universal reaction mechanism (Vorkapić et al., 2026). This in part comes from the diversity of what we mean by biogenic. The reaction may be using purified biomolecules, extracts, oils whole cells or plant parts (Figure 1). The diversity of possible biochemical redox reactions from all possibility of plant and microbe use is quite difficult to define into a list of specific reactions but the general processes would be similar. It is likely that multiple biomolecules and metabolic pathways are likely to contribute simultaneously, with their relative importance depending on the biological system and the metal precursor (Ruotolo et al., 2018). Future mechanistic studies should therefore focus on identifying the dominant reducing and stabilizing components under defined experimental conditions rather than assuming a common mechanism across all biological systems.

### Purification

Purification of the NPs can be a challenging step in biogenic NP production. This process ensures that the synthesized NPs exhibit expected properties. If this stage is not properly conducted, the resulting NPs may contain loosely associated residual biological material, which can alter their characteristics and functionality. Most studies utilizing biogenic approaches overlook a full purification process, leading to conclusions on their material that is not fully understood or appreciated. Studies that conduct purification steps give clearer and more reliable interpretations. It is also important to recognize that purification is not a single objective process. Depending on the intended downstream application, purification may aim to remove unreacted metal ions, soluble biomolecules, loosely associated biological residues, or microbial contaminants, while preserving the tightly bound biomolecular capping layer that contributes to nanoparticle stability and biological activity. Consequently, the definition of a “purified” biogenic nanoparticle depends on the scientific question being addressed. Table 6 overviews purification approaches and possible related concerns.

**TABLE 6:** Examples of Purification approaches of biogenic NPs and their influence.

Purification/separation method(s)	Implication	References
Inadequate Centrifugation and washing	The residual phytochemicals impair characterization and activity	Ahmad et al. (2021)
Dialysis, chromatographic separation	The reagents/surfactants can cause misleading biological responses	Crist et al. (2013)
Filtration/size-based separation emphasized	contamination by the filter matrix and shear force affects can confound ecotoxicity results	Petersen et al. (2014)
Centrifugation, dialysis, standardized washing suggested	Tightly bound biomolecules leads to incomplete removal and complicates interpretation	Singh et al. (2023)
Repeated centrifugation/washing implemented	Purification sharpens spectra and reduces apparent aggregation	Kalantari and Turner (2024)
Multiple wash/centrifuge steps documented	differences in washing methods alter NP morphology and activity	Salayová et al. (2021)

In some studies, extracellular extracts of microorganisms are employed, where purification is primarily achieved through centrifugation (Ojeda et al., 2020; Vetchinkina et al., 2018). However, this process becomes significantly more challenging when NPs are synthesized intracellularly, as an additional cell lysis step is needed. The choice of lysis method will lead to different outcomes. For example, using sonication to break cells can potentially affect NP integrity, whereas chemical agents such as detergents (e.g., Triton X-100 or SDS), which are employed to dissolve cell walls, can become part of the cap of the NP. In other cases, mechanical disruption methods, such as percussion techniques which involve freezing cells or applying high shear forces and/or extrusion through filters or needles of defined size can change the characteristics of the NPs (Piacenza et al., 2019). For higher purity, one can approach a problem similar to that of biochemists aiming to study a protein using chromatography methods, which can be quite successful, but takes time and resources. Likewise, purification methods such as dialysis or ultrafiltration are particularly effective for removing soluble salts, unreacted metal precursors, and small biomolecules, whereas repeated centrifugation and washing are generally more suitable for eliminating loosely associated cellular debris (Khdair et al., 2025; Robertson et al., 2016). No single purification strategy is universally applicable, and the selected method should balance contaminant removal with preservation of the functional biomolecular capping layer. Excessive purification may unintentionally remove relevant surface molecules, whereas insufficient purification may result in misleading characterization and biological activity.

The studies discussed throughout this section consistently demonstrate that purification is not merely a post-synthesis processing step but a fundamental determinant of nanoparticle quality. Insufficient removal of loosely associated biomolecules may alter particle size measurements, surface chemistry, biological activity, and colloidal stability, making comparisons between studies difficult. For biomedical applications, purification may also require the removal of endotoxins and other microbial contaminants that could interfere with biological assays or induce unintended immune responses (Schneier et al., 2020). Establishing standardized purification protocols is therefore essential for improving both reproducibility and the reliability of reported biological effects.

### Characterization

The characterization of NPs is essential for evaluating their functional properties, determining their suitability for specific applications and maintaining reproducibility. This is far more complex for biogenic NPs due to the diversity of biomolecules present. The most commonly used techniques for NP characterization include ultraviolet‒visible (UV‒Vis) absorption spectroscopy, scanning electron microscopy (SEM), transmission electron microscopy (TEM), atomic force microscopy (AFM), Fourier transform infrared spectroscopy (FTIR), X-ray diffraction (XRD), X-ray photoelectron spectroscopy (XPS), and Zeta sizer/dynamic light scattering (DLS). These techniques provide information on size, morphology, surface chemistry, crystallinity, and dispersion stability (Zhang et al., 2016).

#### UV‒Vis Spectroscopy

UV‒Vis spectroscopy is a standard technique for identifying the characteristic surface plasmon resonance (SPR), which confirms the formation of NPs (Burda et al., 2005). Fluorescence spectroscopy is also used to explore possible emission properties as in quantum dots. One factor to consider is the purity of the NPs. UV‒Vis spectroscopy provides distinct absorbance peaks for specific NPs; for example, gold NPs typically exhibit an SPR peak at approximately 500–550 nm (Haiss et al., 2007), whereas silver NPs show a peak near 400 nm (Agustina et al., 2021). However, when biological components are involved in synthesis, their presence with the final NP product interferes with the UV‒Vis results if they are not properly removed during purification and can even mask the NP absorption bands.

Plant extracts or microbial cultures contain high amounts of proteins and nucleic acids. DNA strongly absorbs at 260 nm (Minhas-Khan et al., 2021), whereas proteins absorb from 255 to 295 nm due to the presence of aromatic amino acids, (Biter et al., 2019). Additionally, various cofactors, cytochromes and photopigments absorb 350 to550 overlapping key NP SPR peaks. These molecules may be specifically bound as part of the cap or may be transiently bound and thus can be removed through purification steps. If purification is not properly done the resulting in spectroscopic contamination will confound the interpretation of product.

#### Electron microscopy

SEM is widely used because it produces high-resolution images by scanning the surface of a sample on the nm scale (Harrison et al., 2006; Titus et al., 2019). The majority of NP studies have presented SEM images to confirm their morphology and size (Buhr et al., 2009; Vladár and Hodoroaba, 2020) and, are used for biogenic NPs as well. When NPs are synthesized from biological systems, residual biomolecules can interfere with SEM imaging. If purification is inadequate, biological residues may obscure and cover the NPs, making them difficult to image particularly when the NP concentration is low. In such cases, the NPs may be embedded within organic materials, preventing SEM from effectively capturing their structure. A study carried out in 2010 by Anuradha et al. reported that for gold NPs synthesized via Neem (Anuradha et al., 2010), the SEM images appear unclear due to the presence of biological polymers intertwined with the NPs.

Additionally, for better imaging and stability, SEM samples are often coated with a thin layer of gold or carbon to increase conductivity and reduce charging effects (Rutherford et al., 2024). However, this coating process can sometimes alter the apparent morphology of the NPs, further complicating accurate characterization. This problem is enhanced when the particles are embedded in cellular debris. Finally, the dehydration process of sample prep will change the nature of the cellular components further confounding the interpretation of the images.

TEM has been less used, yet it is useful to obtain the size of the metal core, the presence of biological debris, aggregation, and shape (Presentato et al., 2016). Higher end instruments now allow for electron diffraction that can be used to determine crystallinity (Presentato et al., 2018). There are less issues with sample preparations and the newer instruments require less or no contrast staining. One can also use TEM to observe where in the cell the NP synthesis is taking place, such as cytoplasmic, associated with membranes or cell surface. Regardless both SEM and TEM have energy dispersive X-ray spectroscopy (EDX) for elemental detection (Scimeca et al., 2018).

#### Dynamic light scattering/Zeta sizer

DLS is used to determine the average size and size distribution of NPs (Modena et al., 2019). However, inexperienced users can become confused between the difference in size observed by DLS versus EM. Studies have shown that size estimates from TEM and DLS do not correlate well (Bulgarini et al., 2021; Souza et al., 2016; Yadi et al., 2022). DLS provides the hydrodynamic radius which would be strongly influenced by the biomolecules of the capping layer and presence of cellular debris or even biocoronas. DLS measurement includes the entire NP-biomolecule complex (Figure 2). Furthermore, much of the cellular debris (protein complexes/aggregates, lipid vesicles, carbohydrate or nucleic acid nets) may be within the same size range as the biogenic NPs and will contribute to the measurements. Additionally, the nature of the correlation function lends to bias weighting to larger particles in the suspension. Consequently, DLS-derived sizes are typically an overestimation of the size, as biomolecule layers and further associated biocorona that may not be part of the cap can contribute to the measured hydrodynamic diameter. While DLS may not provide the precise core size of biogenic NPs, it remains valuable for estimating the thickness of the capping layer, loosely associated layers and understanding NP-biomolecule interactions. Most DLS instruments give a size distribution function or provides a poly dispersity index (PDI) to understand how homogenous the sample is.

The Zeta potential is often measured alongside DLS measurement in order to obtain information on the slipping plane potential of the limit of the electric double layer (EDL). The EDL may contain the complete cap of the NP or simply the base. The larger the Zeta potential, typically the more stable the NPs will be and thus will not aggregate (Figure 3).

#### Capping layer molecular analysis

One of the most useful techniques for analysing the molecular capping composition of biogenic NPs is FTIR (Pasieczna-Patkowska et al., 2025). However, when NPs are synthesized via biological methods, particularly in aqueous environments, the high concentration of water molecules can interfere with the spectral readings, leading to distorted results (Kotov et al., 2025). To address this challenge, NPs need to be dried or freeze-dried before analysis to minimize the interference of water molecules (Hoyt et al., 2025) unless there is an SPR of the FTIR available. While FTIR provides valuable insight into the functional groups present on the NP surface cap (Sofyan et al., 2026), determining the exact molecular composition of the capping layer often requires further complementary analyses.

Follow up techniques specific to biogenic NP analysis includes, metabolomic approaches using nuclear magnetic resonance or mass spectrometry to identify the associated small biomolecules present. If proteins are suspected, then proteomics tools can be employed to identify key reductive enzymes that may have been involved in the NP synthesis (Lampis et al., 2017). Lipidomics and genomics approaches can also be applied. Yet, simpler biochemical approaches can also be used to determine relative protein, lipid, carbohydrate concentrations or nucleic acid assays (Bulgarini et al., 2021). Such assays can be quickly performed on a smaller laboratory budget and still provide useful information on the cap’s heterogenous composition (Figure 2).

#### Less used physical chemical approaches for characterizing biogenic nanoparticles

XRD and XPS or even X-ray absorption near edge (XANES) spectroscopy are less frequently used techniques for characterizing biogenic nanoparticles because of their high cost, limited accessibility, and requirement of a synchrotron for advanced analysis. XRD identifies crystalline structures and phase compositions (Holder and Schaak, 2019), but faces challenges when applied to biogenic NPs because of the presence of amorphous biopolymers such as proteins and polysaccharides, which obscure diffraction patterns, leading to weak or broad peaks (Khan et al., 2013). XPS, which provides information on surface chemistry and oxidation states (Baer and Engelhard, 2010), but can also be complicated by thick biomolecule capping layers that can mask signals from the nanoparticle core. Nonconductive organic coatings cause charging effects, leading to peak shifts and inaccurate spectral interpretation. Moreover, the vacuum conditions required for XPS analysis may alter the oxidation state of surface elements, complicating data reliability. Owing to these limitations, XRD and XPS are rarely used for biogenic NP characterization.

AFM is used for characterizing the surface properties of chemogenic nanoparticles, offering high-resolution three-dimensional imaging at the nanoscale (Vahabi et al., 2013). Unlike EM, AFM does not require vacuum conditions or conductive coatings, making it particularly useful for analysing NPs in their hydrated state. This technique can provide information on nanoparticle morphology, surface roughness, and size distribution (Falsafi et al., 2020; Gulati and Adachi, 2023). Unfortunately, the heterogeneous biomolecule organic capping layers, can interfere with imaging. The presence of these organic materials can lead to artefacts, making accurate determination of NP boundaries or measurement of their true surface features difficult. Another challenge lies in the interaction between the AFM tip and soft biofunctionalized nanoparticles. Since AFM operates by scanning a nano tip across the NP surface (Ong and Sokolov, 2007), the applied force can sometimes deform or alter the structure of delicate biological coatings, leading to inaccurate measurements. Furthermore, variations in imaging parameters, such as tip selection, scanning mode, and environmental conditions, can influence the reproducibility and reliability of the results.

Overall, accurate characterization of biologically synthesized nanoparticles requires the combined use of complementary chemical and biochemical analytical techniques, as each method provides different information and is affected differently by the biomolecular capping layer and residual biological materials. The major strengths and limitations of the commonly used characterization techniques are summarized in Table 7.

**TABLE 7:** Influence of biomolecular capping layers and biological residues on the characterization of biogenic nanoparticles.

Characterization technique	Primary information obtained	Effect of biomolecular cap/biological residues	Practical implication
UV–Vis	Surface plasmon resonance	Proteins, pigments and nucleic acids introduce overlapping absorbance	Difficult confirmation of NP formation
SEM	Morphology and particle size	Organic residues can embed or cover nanoparticles	Poor image quality and inaccurate size estimation
TEM	Core size and morphology	Capping layer and debris obscure particle boundaries	Core easier to visualize than SEM but interpretation still affected
DLS	Hydrodynamic diameter	Measures the entire NP + cap + loosely bound biomolecules	Particle size usually overestimated
Zeta potential	Surface charge	Cap composition changes measured surface charge	Stability comparisons become difficult
FTIR	Functional groups	Water and heterogeneous biomolecules produce overlapping bands	Difficult identification of capping molecules
XRD	Crystallinity	Amorphous biomolecules weaken diffraction peaks	Reduced crystal identification
XPS	Surface chemistry	Thick organic cap masks metal surface	Reduced sensitivity to metal core chemistry
AFM	Surface topography	Soft biomolecular coatings deform during scanning	Reduced measurement accuracy

**TABLE 8:** Minimum reporting checklist for studies on biologically synthesized nanoparticles.

Stage	Recommended information to report	Why it is important
Biological source	Species, strain/cultivar, plant part or microorganism, source/location	Improves reproducibility
Growth/collection conditions	Season, cultivation conditions, growth phase, culture medium	Biological composition varies
Extract preparation	Solvent, extraction time, temperature, extract concentration	Determines reducing/capping molecules
Metal precursor	Metal salt, concentration, purity	Influences nucleation
Reaction conditions	pH, temperature, reaction time, mixing, precursor/extract ratio	Controls NP formation
Yield	Nanoparticle yield or conversion efficiency	Enables process comparison
Purification	Centrifugation, washing, dialysis, filtration, chromatography	Removes unbound biomolecules
Storage	Temperature, storage medium, duration	Influences stability
Size characterization	At least two complementary techniques (e.g., TEM + DLS)	Distinguishes core size from hydrodynamic diameter
Crystallinity	XRD or electron diffraction	Confirms crystal structure
Elemental composition	EDX, ICP-OES, ICP-MS or equivalent	Confirms elemental composition
Surface chemistry	FTIR, XPS, proteomics/metabolomics where appropriate	Characterizes capping layer
Colloidal stability	Zeta potential and stability over time	Evaluates dispersion stability
Contaminant assessment	Residual biomolecules, endotoxin (for microbial synthesis), residual metal salts	Avoids misleading biological results
Biological controls	Extract-only control, metal salt control, chemically synthesized NP control where appropriate	Distinguishes NP effects from synthesis components

## Challenges in using biogenic NPs for *in vivo* medical applications

Biogenic NPs have demonstrated strong potential in various biomedical applications, ranging from drug delivery and cancer therapy to antimicrobial treatments and vaccine development (Malik et al., 2023). For example, biogenic silver NPs have demonstrated anticancer properties, showing potential for targeted cancer treatment and therapeutic applications (Kah et al., 2023). However, their practical implementation faces multiple challenges, particularly concerning stability, reproducibility, biological interactions, and functional efficiency. Below, we discuss the challenges associated with specific *in vivo* applications.

### Drug delivery

In drug delivery applications, maintaining NP stability is essential for ensuring biogenic NPs often suffer from poor stability due to variations in surface charge and capping layer composition between particles produced, which occurs from the biological diversity biological sources such as plants, bacteria, and fungi, as well as synthesis conditions and culture environments. An unstable or distributed surface charge can lead to aggregation in physiological fluids, reducing bioavailability and impairing drug delivery efficiency (Xu et al., 2018). Additionally, the diversity of biological molecules from the synthesis process introduces batch-to-batch variability, making it difficult to meet the regulatory standards required for drug formulations. Furthermore, the lack of precise control over NP size and shape can significantly affect the drug loading capacity and release kinetics, leading to unpredictable therapeutic outcomes. Although biogenic NPs show promise for drug delivery (Abdel-Megeed, 2025), particularly if matching the synthesis biology to the drug development there has been little done on the pharmacokinetics biogenic vs. chemogenic NPs. In addition, the adsorption of plasma proteins following systemic administration may alter the physicochemical properties of biogenic nanoparticles, influencing their biodistribution, cellular uptake, circulation time, and clearance by the mononuclear phagocyte system. These interactions are highly dependent on the composition of the biomolecular capping layer, which may differ considerably among synthesis methods. As a result, nanoparticles with similar core sizes may exhibit different *in vivo* behaviours, making it difficult to predict therapeutic performance. These challenges highlight the need for carefully defined synthesis techniques and rigorous quality control measures to increase the reliability of biogenic NPs for drug delivery applications.

### Cancer therapy

In cancer therapy, the size homogeneity of the NPs and the formation of a biocorona play roles in their ability to effectively target tumors and circulation residency times. The enhanced permeability and retention (EPR) effect favours NPs smaller than 50 nm, as they exhibit better tumour accumulation, whereas larger particles are more susceptible to clearance by the mononuclear phagocyte system (MPS) (Ejigah et al., 2022; Park et al., 2019). However, biologically synthesized NPs often display a broad size distribution, and often larger due to the bimolecular caps, which can reduce their targeting efficiency and limit their therapeutic potential. Additionally, the biocorona effect, caused by the adsorption of host proteins and biomolecules onto the biogenic NP surface in the bodies various environments, may alter NP‒cell interactions, leading to reduced specificity and lower uptake by cancer cells. Furthermore, the molecular composition of the capping layer can contribute to unexpected immune recognition or off-target accumulation, potentially compromising the efficacy and safety of NP-based cancer therapies. Addressing these challenges requires greater control over NP synthesis parameters and effective reproducible purification approaches.

### Antimicrobial applications

As we enter the antimicrobial resistance (AMR) era, where antibiotic-resistant bacteria have become increasingly prevalent due to both misuse and overuse of antibiotics, there is an urgent need for new strategies to combat these resistant strains. NPs offer promising potential as antibacterial agents to address this critical issue (Turner, 2024). NPs synthesized via biogenic means exhibit improved drug characteristics, such as greater stability and enhanced antimicrobial activity, compared with those produced through chemical/physical methods (Lashani et al., 2024; Piacenza et al., 2017; Piacenza et al., 2018). It is accepted in the literature that biogenic NPs can exhibit strong antimicrobial properties, making them candidates for combating antibiotic-resistant infections (Kalantari and Turner, 2024). However, the biological synthesis process introduces potential confounding factors that complicate the accurate evaluation of their antimicrobial activity. Residual plant/microbial metabolites from the synthesis process or from insufficient purification may contribute to the observed effects, leading inaccurate understanding of the therapeutic active compound. Many studies fail to distinguish between the true antimicrobial action of NPs and the inherent activity of these residual compounds (Franco-Castillo et al., 2021; Turner, 2024). This issue is particularly important when plant extracts containing phenolics, flavonoids, terpenoids, or antimicrobial peptides are used during synthesis, as these compounds may remain associated with the nanoparticles even after purification. Consequently, the reported antimicrobial activity may reflect the combined effects of the nanoparticle and residual biomolecules rather than the nanoparticle alone. Additionally, organic molecules present in the capping layer can influence NP interactions with bacterial membranes, making it challenging to compare antimicrobial efficacy across different studies. In practical applications such as wound dressings, surface coatings, and injectable formulations, the stability of biogenic NPs in complex biological environments is a concern, as aggregation may reduce their antimicrobial efficiency and limit their real-world effectiveness. Addressing these challenges requires improved purification methods and standardized characterization protocols to ensure accurate assessments of NP antimicrobial properties and bioactive components.

### Imaging and diagnostics

Nanoparticles have been investigated as contrast agents for medical imaging because of their unique optical and electronic properties (Aslan et al., 2020). However, biogenic NPs effectiveness is often hindered by challenges related to surface composition and functionalization. Variability in the capping layer composition can lead to inconsistent attachment of targeting ligands, reducing specificity in imaging applications. Additionally, NP stability in physiological fluids is crucial for maintaining signal clarity, as many biogenic NPs are prone to degradation or aggregation compromising their imaging performance. Another important consideration is that adsorption of serum proteins following administration may modify the optical and surface properties of biogenic nanoparticles, thereby altering imaging sensitivity, biodistribution, and target specificity. These dynamic biological interactions remain insufficiently understood and require further investigation before routine clinical application can be achieved.

### Vaccine development

Nanoparticles are being explored as adjuvants in vaccine development to enhance immune responses (Kheirollahpour et al., 2020). However, the use of biogenic NPs raises several questions regarding their immunogenicity due to other biomolecules in their cap, reproducibility, and long-term safety. Biological residues from plant or microbial synthesis could trigger unintended immune responses, complicating the formulation of vaccines. Additionally, batch-to-batch variability in the surface composition may lead to inconsistent immune activation, which could affect the overall efficacy of the vaccine. Furthermore, the long-term biocompatibility, biodistribution, and clearance mechanisms of biogenic NPs, are raising concerns about potential toxicity and the risk of accumulation in organs over time. Another challenge is that the biomolecular capping layer may contain structurally diverse proteins, polysaccharides, or other metabolites that influence antigen presentation and immune-cell recognition. While this may enhance adjuvant activity in some cases, it may also increase variability in immune responses, making standardization and regulatory approval more difficult. Consequently, comprehensive *in vivo* studies evaluating biodistribution, biodegradation, immunogenicity, and long-term safety remain necessary before the widespread clinical translation of biogenic nanoparticle-based vaccines.

## Environmental risks and safety

### Green chemistry

Many studies producing NPs suing biogenic approaches express strongly that the process is Green or at least more eco-friendly. For the chemistry to be green the key goal is to reduce and/or eliminate the use and production of harmful substances. To do this, one often thinks to use renewable materials in which plant and microbial sources fit well. An important consideration is the amount of energy consumption and production of greenhouse gases. This is often overlooked when doing biogenic synthesis. For example, research conducted by Patra et al. (Patra et al., 2015) and Nadaouda et al. (Nadagouda et al., 2014) used *Butea monosperma* (blackberry) extracts for gold NP synthesis. However, the process requires an overnight reaction time to increase yields, resulting in increased energy costs. If using plants how much fertilizer and energy of harvest? Energy for preparing the extract. Similarly, using a microbial fermenter will use a lot of energy for temperature and mixing control. Even though higher yields can be obtained, should we really consider biogenic production “Green”. Compared to chemical or physical methods they may be somewhat more eco-friendly, but this can be also lost if one does not consider use and disposal problems. Overall such monikers may be stretching the truth for marketing purposes.

### Environmental fate and bioaccumulation

With the widespread use of all nano materials, concerns about their environmental persistence and accumulation have intensified (Kumah et al., 2023). Regardless of their intended application, NPs inevitably enter ecosystems through industrial discharge, agricultural runoff, and through wastewater treatment plants (WWTP). Current wastewater treatment technologies are not specifically designed to remove nanoparticles, leading to their uncontrolled release into aquatic and marine environments. The extent of nanoparticle removal during wastewater treatment varies considerably depending on particle composition, size, surface chemistry, and treatment technology. Consequently, a fraction of nanoparticles remains in treated effluents, while a substantial proportion becomes associated with sewage sludge, creating additional routes for environmental exposure when biosolids are applied to agricultural land. Once introduced, NPs interact with sediments, microorganisms, and aquatic/marine organisms, resulting in bioaccumulation through food chains. Studies have shown that prolonged exposure to high concentrations of NPs can negatively impact plant growth particularly when excessive loads are introduced (Dey and Nandy, 2024). Reported effects include reduced seed germination, inhibition of root elongation, decreased chlorophyll content, and altered nutrient uptake, although these responses vary according to nanoparticle composition, concentration, exposure duration, and plant species (Djanaguiraman et al., 2024; Siddiqi and Husen, 2022). The accumulation of NPs in plants is especially concerning, as plants serve as a primary food source for higher organisms, extending the risks. Several studies (Fetouh et al., 2024; Sharma et al., 2024; Sivakumar et al., 2025) have demonstrated that NPs exhibit toxicity in aquatic organisms, disrupting essential biological processes such as respiration, reproduction, and metabolic function. A common mechanism of toxicity involves the generation of reactive oxygen species, which may result from direct nanoparticle-cell interactions, the release of dissolved metal ions, or a combination of both, depending on the physicochemical properties of the nanoparticles. The resulting oxidative stress induces lipid peroxidation and DNA damage in aquatic species, including fish and algae (Bacchetta et al., 2017; Yu et al., 2020). Oxidative stress may further impair mitochondrial function, activate inflammatory signalling pathways, and induce programmed cell death, thereby affecting growth, reproduction, and survival even when no immediate mortality is observed. Even at low concentrations, biogenic silver nanoparticles have been shown to alter oxidative biomarkers and impair reproductive success in fish, underscoring that sublethal exposure can still disrupt population health (Valerio-García et al., 2017). Of course, size and surface characteristics play roles, with smaller NPs generally exhibiting greater bioavailability and toxicity due to greater surface reactivity and cellular uptake (Naz et al., 2020; Phan et al., 2020). Surface charge, particle dissolution, and the release of metal ions may also contribute to toxicity, particularly for silver- and zinc-based nanoparticles, where distinguishing nanoparticle-specific effects from dissolved-ion effects remains an important challenge when interpreting environmental toxicity studies (Attarilar et al., 2020; Cho et al., 2012). These physicochemical factors also influence environmental interactions. For example, in soils and aquatic sediments, ZnO and other metal-oxide NPs have been found to inhibit nitrifying bacteria and alter the microbial community composition, thereby impairing nitrogen cycling and soil fertility (Chen et al., 2021; Priester et al., 2012). Because these microbial communities regulate key biogeochemical cycles, prolonged disruption may ultimately influence nutrient availability and ecosystem productivity.

Although biogenic NPs are often considered safer than their chemically synthesized counterparts because of their ‘biocompatible’ capping agents, their toxicity still remains dose- and context dependent. While some biogenic NPs show reduced acute toxicity, high concentrations or chronic exposure still pose risks to organisms and ecosystems (Limbach et al., 2008; Mohana et al., 2021). Furthermore, environmental transformation processes such as oxidation, dissolution, aggregation, and interaction with natural organic matter may substantially modify nanoparticle behaviour after release, meaning that toxicity observed immediately after synthesis may differ from that occurring following environmental ageing. Moreover, WWTPs are unable to completely remove these NPs, leading to their release into effluents and sludge, from which they can contaminate underground water systems and enter agricultural irrigation pathways (Sharma et al., 2020; Wheeler et al., 2021). In terrestrial environments, plant exposure to NPs raises additional concerns. Greenhouse studies with soybean have shown that nano-ZnO and nano-CeO_2_ not only alter plant growth and nutrient uptake but also disturb the root-associated microbiota, indicating a risk of food-chain transfer and bioaccumulation (Ge et al., 2014; Priester et al., 2017; Reichman et al., 2024). An important consideration when interpreting environmental studies is distinguishing the effects of intact nanoparticles from those arising from dissolved metal ions released during particle transformation or dissolution (Lead et al., 2018). Depending on the nanoparticle composition and surrounding environmental conditions, both mechanisms may contribute to the observed biological responses, and their relative importance can vary considerably among different nanoparticle systems (Auría-Soro et al., 2019). Such changes in rhizosphere microbial communities may indirectly influence plant health by altering nutrient cycling, root colonization, and resistance to environmental stress. Furthermore, the formation of a biocorona or eco-corona modifies their identity, persistence, and uptake, making it challenging to predict their true environmental behaviour (Devasena et al., 2022; Wang et al., 2022). Taken together, these findings highlight that the ecological toxicity of biogenic NPs is driven by both intrinsic factors (size, surface chemistry, and dose) and extrinsic factors (environmental chemical/geological matrices and biocorona formation). Current knowledge is still largely derived from short-term laboratory studies conducted under controlled conditions, whereas considerably fewer investigations have examined chronic exposure, environmentally realistic concentrations, or complex natural ecosystems. This underscores the need for mechanistic and long-term risk assessments of biogenic NPs in the environment, before large-scale applications of biogenic NPs can be considered safe and become routine.

### Safety in storage and handling

To mitigate unintended environmental exposure, strict protocols for the storage and handling of NPs should be carefully controlled. Although regulations are mostly in place for various nanomaterials, biogenic NPs are falsely considered safer as they are ‘green’. Although they may be green or eco-friendly in synthesis they are not necessarily safe in applications. Given their potential toxicity, biogenic NPs should be stored in controlled environments to prevent accidental spills and airborne contamination (Amoabediny et al., 2009). Failure to regulate biogenic NP handling can lead to environmental contamination, once airborne the could easily disperse, infiltrating both indoor and outdoor environments. Furthermore, biogenic metal NPs when they degrade of course release potentially toxic metals into the environments (Cerwenk et al., 1961; Jain et al., 2020; Vávrová et al., 2024).

### Biosafety concerns

The use of microorganisms for biogenic NP introduces additional biosafety concerns. Certain bacterial and fungal species utilized in NP production may be pathogenic, increasing the risk of biological contamination. Importantly, the presence of viable microorganisms, residual microbial components, and endotoxins should be considered as separate biosafety concerns (Ustundag and Ustundag, 2026). Even when microbial cells are completely removed during purification, immune-reactive components such as lipopolysaccharides from Gram-negative bacteria or other cell-wall constituents may remain associated with the nanoparticle preparation and influence biological responses (Gan et al., 2023). Therefore, sterilization alone does not necessarily ensure endotoxin-free nanoparticle formulations, highlighting the importance of appropriate purification and quality control procedures, particularly for biomedical applications. Although endotoxin detection assays such as the Limulus Amebocyte Lysate (LAL) assay are used for quality control, nanoparticles may interfere with these assays through adsorption of assay components or optical interference, potentially resulting in inaccurate endotoxin measurements (Li et al., 2017). If not handled properly, the microorganisms used in synthesis could be unintentionally released into the environment, disrupting native microbial communities or even posing a direct threat to human and animal health. The metabolic byproducts of microbial synthesis may interact with NPs, altering their physicochemical properties and environmental behaviour (Eduok and Coulon, 2017). This unpredictability raises concerns about long-term ecological impact and necessitates the implementation of rigorous sterilization and containment protocols.

The reporting checklist provided in Table 8 covers key parameters for storage, purification, and contamination assessment to support safer and more transparent research practices. Overall, current evidence indicates that the environmental behaviour of biogenic nanoparticles is governed not only by their metallic core but also by their evolving biological surface. Environmental transformations, including interactions with natural organic matter and the formation of eco-coronas, can substantially modify nanoparticle transport, persistence, bioavailability, and toxicity. Consequently, environmental risk assessments should consider nanoparticles as dynamic systems whose properties change continuously after environmental release rather than as static materials characterized only immediately after synthesis.

## Conclusion

Biological synthesis has emerged as an alternative to conventional chemical and physical methods for nanoparticle production because it offers more sustainable, environmentally friendly, and potentially biocompatible approaches. Nevertheless, as discussed throughout this review, the widespread application of biogenic nanoparticles is still constrained by several important challenges. Variability in biological sources, limited control over synthesis conditions, inconsistent purification procedures, and difficulties in accurately characterizing nanoparticles with complex biomolecular capping layers all contribute to poor reproducibility and hinder meaningful comparisons among studies. Furthermore, incomplete understanding of nanoparticle–biomolecule interactions, long-term stability, *in vivo* behavior, and environmental fate continues to limit their translation into reliable biomedical and industrial applications.

Overcoming these limitations will require the development of standardized and reproducible protocols for nanoparticle synthesis, purification, and characterization. Greater attention should also be directed toward understanding the molecular composition and biological functions of the capping layer and the dynamic formation of biocoronas, as these factors strongly influence nanoparticle stability, biological activity, cellular interactions, and environmental behavior. Equally important is the implementation of comprehensive *in vivo* and environmental risk assessments that evaluate biodistribution, biodegradation, long-term toxicity, bioaccumulation, and ecological impacts under realistic exposure conditions. Such studies will be essential for establishing robust regulatory frameworks and ensuring the safe development of biogenic nanotechnologies.

Overall, the future of biogenic nanoparticles extends beyond simply replacing conventional chemical or physical synthesis methods. Their successful translation into clinically and industrially relevant technologies will depend on integrating sustainable production with rigorous purification, comprehensive physicochemical characterization, standardized methodologies, and thorough safety evaluation. This review is by no means comprehensive and does not consider the issues of at scale manufacturing and engineering regulations and frame works. So few studies and reports exist for the steps at the end of the schema of Figure 1, and we hope this review will bring them attention for future research. Regardless addressing the research challenges herein will not only improve the reliability and reproducibility of biogenic nanoparticle research but also accelerate the responsible development of safe and effective nanomaterials for biomedical, environmental, and industrial applications.
